# Personal

**Published:** 1898-08

**Authors:** 


### PERSONAL.

Dr. Charles A. L. Reed, of Cincinnati, delivered the doctorate address at the joint commencement exercises of the Louisville College of Dentistry and the Hospital College of Medicine, departments of Central University of Kentucky, held at Macauley's theatre, Louisville, June 30, 1898. It is needless to add that Dr. Reed was at his best on this occasion before the brilliant audience assembled and that his address was eloquent, thoughtful and instructive. After the exercises the faculty entertained Dr. Reed at dinner at the Pendennis club.

---

Dr. L. S. McMurtry, of Louisville, Ky., has been elected president of the faculties of the Hospital Medical College and the Louisville College of Dentistry, vice Dr. John A. Larrabee, deceased. Dr. McMurtry is professor of gynecology and abdominal surgery in the first named college and is everywhere recognised as one of the foremost

American pelvic and abdominal surgeons. His election to the presidency will add further luster to an already famous institution.

---

Dr. William H. Draper tendered his resignation as professor of clinical medicine in the College of Physicians and Surgeons—medical department of Columbia University—to take effect June 30, 1898. Dr. Draper was appointed professor in 1869 and has served the University since that time. In recognition of his services the board of trustees voted to make him an emeritus professor for life.

---

Dr. Vertner Kenerson, captain and assistant surgeon of the 74th, has been appointed a contract surgeon in the army and has departed for Washington, where he will report to Surgeon-General Sternberg. He expects to be ordered to the United States general hospital at Tampa.

---

Dr. Charles P. Knowles, a graduate of the College of Physicians and Surgeons at New York, has located at Buffalo. His office and residence are at 878 Elmwood avenue. Hours: 8-9 a. m., 2-3 and 7-8 p. m. Telephone, Bryant 2,001.

---

Dr. Henry D. Ingraham, of Buffalo, has resigned as gynecologist at the Buffalo Hospital of the Sisters of Charity and is now connected with The Riverside, a private hospital, located at 306 Lafayette avenue, Buffalo, N. Y.

